# Anticancer Properties of Different Solvent Extracts of *Cucumis melo* L. Seeds and Whole Fruit and Their Metabolite Profiling Using HPLC and GC-MS

**DOI:** 10.1155/2020/5282949

**Published:** 2020-02-24

**Authors:** Xudong Zhang, Yuzhuo Bai, Yun Wang, Chunlan Wang, Jianhua Fu, Longlan Gao, Yu Liu, Jingbin Feng, Mallappa Kumara Swamy, Maddipatla Yogi, Gudepalya Renukaiah Rudramurthy, Boregowda Purushotham, Yue Deng

**Affiliations:** ^1^Encephalopathy Center, The Affiliated Hospital to Changchun University of Chinese Medicine, Changchun, Jilin Province 130021, China; ^2^Extrathoracic and Thyroid Mammary Surgery, The Affiliated Hospital to Changchun University of Chinese Medicine, Changchun, Jilin Province 130021, China; ^3^Department of Respiratory, First Clinical College of Chinese Academy of Traditional Chinese Medicine, Changchun City, Jilin Province 130021, China; ^4^Reproductive Center, Jilin Provincial People's Hospital, Changchun, Jilin Province 130021, China; ^5^Brain Surgery, Liaoyuan Hospital of Traditional Chinese Medicine, Liaoyuan, Jilin Province 136000, China; ^6^Department of Orthopedics, People's Hospital, Sanya, Hainan Province 572000, China; ^7^Department of Biotechnology, East West First Grade College of Science, Bengaluru 560091, India; ^8^Heart Disease Center, The Affiliated Hospital to Changchun University of Chinese Medicine, Changchun, Jilin Province 130021, China

## Abstract

Honeydew melon (*Cucumis melo* L.) is an oval-shaped delicious fruit of one cultivar group of the muskmelon with immense nutritional importance and is extensively consumed by many tropical countries. The effect of various organic solvents on the recovery of phytochemicals from honeydew melon plant fruits and seeds was assessed. Further, High-Performance Liquid Chromatography (HPLC) was used to examine and assess the contents of phenolic acid (gallic acid) and flavonoid (rutin) compounds. The use of gas chromatography–mass spectrometry (GC-MS) analysis explained the presence of volatile phytocompounds in the extracts. The use of organic solvents had a substantial impact on the total dry weight and extract yield. In general, the solvent-extracted constituents remained in the order of methanol>chloroform>distilled water for both honeydew melon seeds and whole fruit. 3-(4,5-Dimethylthiazol-2-yl)-2,5-diphenyl tetrazolium bromide (MTT) was used to assess the cytotoxicity effect against PC3, HCT116, HeLa, and Jurkat cell lines. The chloroform extract exhibited a good cytotoxic activity against all cell lines as compared to other solvent extracts. HPLC analysis revealed the occurrence of gallic acid content of 0.102 ± 0.23 mg/10 mg of dry whole fruit extract, while 10 mg of dry seed extract contained only 0.022 ± 0.12 mg of gallic acid content. Likewise, rutin content was observed to be 0.224 ± 0.31 mg and 0.1916 ± 0.82 mg/10 mg of dry whole fruit and seed extract, respectively. Further, GC-MS analysis revealed the presence of a total of 37 compounds in chloroform extract of whole fruit, while only 14 compounds were found in seed extract. Nevertheless, more examinations are needed to identify and characterize other metabolites from honeydew melon and evaluate their pharmacological importance.

## 1. Introduction

Natural products play a significant role in the modern medicine. Ever since ancient period, naturally derived plant products are being widely used by mankind for treating several ailments. Nature has existed as a source of almost all drugs for many years, and herb-based products play an important role in primary human health care as the majority (80%) of the global population rely on traditional medical practices [1, 2]. Several drugs used in the current medicinal practices are mainly derived either from natural resources or from their chemical derivatives. Also, plant-derived phytocompounds are having huge demand in the pharmaceutical industry [2]. Currently, the prevailing health issues along with emergence of communicable infections and disorders are a major concern to the world population as they are seriously causing increased mortality rate day by day. About 50% of all mortality arising in nations is because of these diseases [3, 4]. Hence, a search for an effective curative agent is continuous. In this regard, natural products are the best choice of sources to isolate and identify new leads for discovering novel drug molecules. This is due to the fact that natural products are cost-effective, exhibit therapeutic potential with high efficacy, and possess no or negligible toxicity effect. The available traditional information related to the therapeutic importance of plants is the basis for their explorations by the experts [5].

Orange-fleshed honeydew melon, also known as (*Cucumis melo* L.), is an oval-shaped delicious fruit with immense nutritional importance and is extensively consumed by many tropical countries. It is one cultivar group of the muskmelon and belongs to the family, *Cucurbitaceae*. It is produced by crossing orange-fleshed cantaloupe with non-netted, green-fleshed honeydew [6–9]. The distinctive aroma of orange-fleshed honeydew melon fruit is due to the presence of several volatile compounds that are derived biosynthetically from fatty acids, amino acids, carotenoids, and terpenes. The major volatile constituents imparting include phenylethyl alcohol and (*Z*,*Z*)-3,6-nonadien-1-ol. The melon fruit is a good source of vital vitamins, i.e., thiamine, riboflavin, and folic acid; provitamin A; and ascorbic acid [7, 9]. It is stated that different parts of the orange-fleshed honeydew melon fruit possess varied levels of soluble solids, total sugars, sucrose, 5-methyltetrahydrofolic acid, and *β*-carotene [6]. Studies have revealed that secondary metabolites accumulated in plant species depend on different parameters including plant organs and ecological conditions [10, 11]. Also, the amount of the phytocompounds isolation via different extraction approaches may significantly fluctuate. Further, different solvents used for extracting plant-derived compounds can influence on the yield of phytocompounds [11]. Literature survey shows that meager information is available on phytochemicals and bioactivities of honeydew melon plant's different organs, and there is no comparative studies related to this aspect. Therefore, in this study, the effect of various organic solvents on the recovery of phytochemicals from honeydew melon plant fruits and seeds is assessed. Anticancer activities were assessed to compare between fruit and seed extracts. Further, High-Performance Liquid Chromatography (HPLC) was used to examine and assess the total phenolic content. The use of gas chromatography–mass spectrometry (GC-MS) analysis explained the presence of volatile phytocompounds in the extracts.

## 2. Materials and Methods

### 2.1. Collection of Honeydew Melon Fruits

Five completely ripened honeydew melon (*Cucumis melo* L.) fruits were collected directly from the agricultural field during the harvesting season (August, 2018). The fruits were then washed to remove the dust particles and then wiped neatly with sterile cotton cloth to remove any traces of water on the surface. Later, the fruits were cut open to obtain the seeds, and one whole fruit was cut in to pieces without removing any contents. The samples were then dried in a hot air oven till they were completely dried at 55°C–65°C and then ground to powder using a blender. All the powdered samples were then weighed and kept in air-tight containers for further use.

### 2.2. Solvent Extraction of Phytochemicals

The phytochemicals from powdered seeds and whole fruits were extracted using three different solvent systems such as distilled water, chloroform, and methanol to determine the ideal solvent extraction system. Each dried powdered sample weighing 20 g was added into 300 mL solvents such as distilled water, chloroform, and methanol in a conical flask and stirred for 24 h. Later, the solutions were double filtered using Whatman filter No.1 filter paper, and the filtrates were then evaporated completely in incubator at 40°C. After complete evaporation, the extracts were weighed, and the extract yield (%) and total dry weight were determined. Later, the dried extracts were preserved at 4°C in for further use.

### 2.3. Cytotoxic Assay through MTT Method

The cytotoxic activity of phytoextracts was determined by 3-(4,5-dimethylthiazol-2-yl)-2, 5-diphenyl tetrazolium bromide (MTT) reduction assay. The cytotoxic activity of different extracts was determined against human prostate cancer cell line (PC-3), human colon cancer cell line (HCT116), immortalized human T lymphocyte cells (Jurkat), and immortal cell line (HeLa). For MTT assay, cell lines were seeded in 96 well culture plates, at a cell density of 20,000 cells per well (200 *μ*L cell suspension), without the test agent and allowed to grow for about 12 h. The IC_50_ values of different phytoextracts against these cell lines were determined using the concentrations 50, 150, 250, 350, and 450 *μ*g/mL, and the assay was carried out in triplicates. The culture medium without cells was used as medium control, whereas, medium with cells, but without the experimental drug/compound, was used as negative control, and medium with cell lines containing curcumin (15 *μ*M) was used as a positive control. All the plates were incubated for 24 h at 37°C in humidified CO_2_ (5%) incubator. After the incubation period, the plates were taken out from incubator, and spent media was removed followed by the addition of MTT reagent to a final concentration of 0.5 mg/mL. The plates were returned to the incubator and incubate for 3 h and then the MTT reagent was removed, and 100 *μ*L of solubilization solution (DMSO) was added. The absorbance was in an ELISA reader at 570 nm with reference wavelength 630 nm. The IC_50_ values for each extract were determined by using linear regression equation, i.e., *Y* = *mX* + *C*. Here, *Y* = 50, *m* (angular coefficient) and *C* (linear coefficient) values were derived from the viability graph, by the relationship between extract concentration and absorbance (cell viability).

### 2.4. High-Performance Liquid Chromatography (HPLC) Analysis of Polyphenols

The analysis was made in isocratic mode with the mobile phase acetonitrile and water in the ratio 7 : 3 with the RP-HPLC C-18 column at a flow rate of 1 mL/min. The standards, gallic acid and rutin (Sigma Aldrich, St. Louis, MO, USA) (0.4 mg/mL), and samples (10 mg/mL) were dissolved in mobile phase, and 20 *μ*L was injected, and the elution was monitored. The spectral data was obtained between 190 and 500 nm. The available standards, their UV spectra, and retention times were compared with the samples to identify and quantify phenolic compounds.

### 2.5. Gas Chromatography–Mass Spectrometry (GC-MS) Analysis

Each solvent extract was subjected to GC-MS analysis using the model instrument, GCMS-QP2010 Ultra (Shimadzu Co., Japan) attached with a capillary column DB-1 (0.25 *μ*m film × 0.25 mm I.d.×30 m length). Analysis was performed by injecting 1 *μ*L of the sample with a split ratio of 20 : 1, and Helium gas (99.9%) was used as the carrier gas at a flow rate of 1 mL/min. The analysis was performed in the electron impact (EI) mode with 70 eV of ionization energy. The injector temperature was maintained at 250°C (constant). The column oven temperature was set at 50°C (held for 3 min), raised at 10°C per min to 280°C (held for 3 min) and finally held at 300°C for 10 min. The compounds were identified after comparing the spectral configurations obtained with that of available mass spectral database (NIST and WILEY libraries).

### 2.6. Statistical Analysis

The results were statistically analyzed using GraphPad Prism (version 5.0) statistical software and expressed as the mean ± standard deviation (SD). Multiple comparisons were done through Dunnett's test by comparing the test data with control/untreated data to determine the statistically significant differences. Further, Student's *t*-test was used to compare the cytotoxicity effect between whole fruit and seed extracts. *p* value ≤0.05 was considered statistically significant.

## 3. Results

The use of organic solvents had a substantial impact on the total dry weight and extracts yield. Comparatively, the methanolic whole fruit extract showed with a higher extraction yield of 11.12%, and total dry weight was observed to be 546.01 ± 1.91 *μ*g of dry weight. Likewise, the methanolic seed extract showed the highest extraction yield of 11.62% with 552.02 ± 2.23 *μ*g of total dry weight. The water extract of both seeds and whole fruit yielded the lowest dry weight and yield. In general, the solvent-extracted constituents remained in the order of methanol>chloroform>distilled water for both honeydew melon seeds and whole fruit.

**Figure 1:**
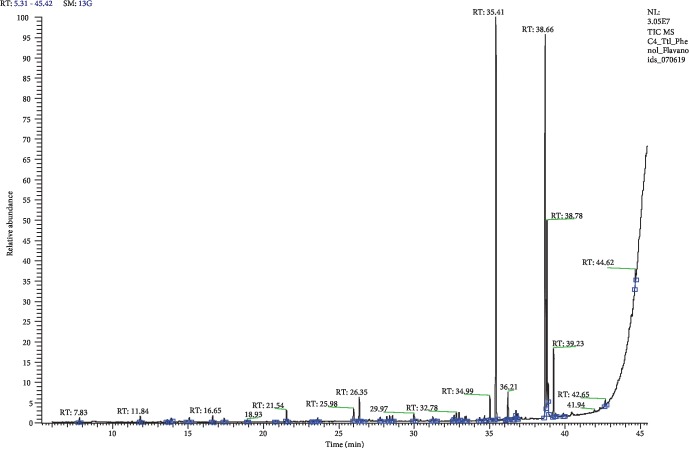
The major phytocompounds detected in the chloroform whole fruit extract of honeydew melon using GC-MS analysis.

The HPLC analysis used to identify the presence of polyphenols in the chloroform extracts of seeds and whole fruit showed the maximum antiproliferative activity against all the cells. The gallic acid content was found to be 0.102 ± 0.23 mg/10 mg of dry whole fruit extract, while for seeds, it was observed to be 0.022 ± 0.12 mg/10 mg. Likewise, the flavonoid, rutin, was observed to be 0.224 ± 0.31 mg/10 mg of dry whole fruit extract. In the chloroform extract of seeds, it was found to be 0.1916 ± 0.82 mg/10 mg. Further, the GC-MS analysis revealed that the chloroform extract of whole fruit contains a total of 37 compounds, while seed extract contained only 14 compounds. The identified nonvolatile compounds of chloroform whole fruit extracts are given in Table 1 and Figure 1.

To explore the cytotoxicity effect of methanol, chloroform and water extracts of seeds and whole fruit of honeydew melon were determined by MTT assay. The anticancer activity was carried out against different cancer cells, such as PC3, HCT116, HeLa, and Jurkat cell lines, and the results showed a decreased viability (%) with increased concentration of the phytoextracts. The results have shown that different solvent extracts of whole fruit and seeds inhibited different cancer cells in a dose-dependent manner. The methanol and chloroform extracts exhibited a good cytotoxic activity against HeLa cell lines as compared to distilled water extracts (Table 2).

**Figure 2:**
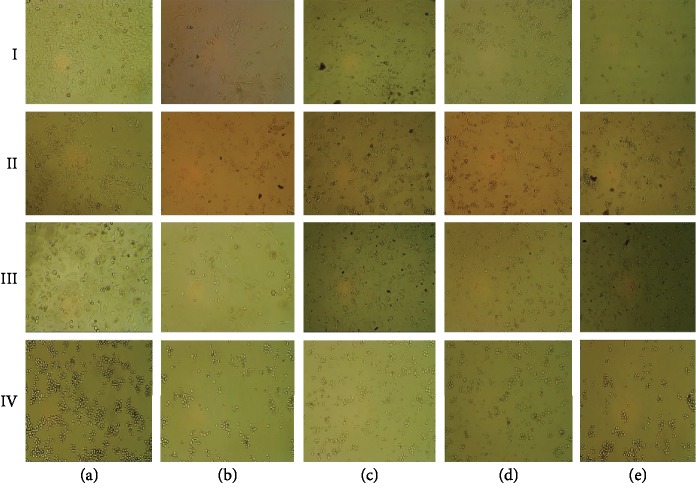
Anticancer activity of honeydew melon whole fruit extract against different cancer cell lines, (a) untreated/control HeLa cell line, (b) treated with curcumin (15 *μ*M), and (c–e) treated with methanol, water, and chloroform seed extracts, respectively, at IC_50_ values. I, II, III, and IV: HeLa, HCT116, PC3, and Jurkat cell lines, respectively.

However, almost similar cytotoxic activity has been exhibited against HCT116 by all different solvent extracts tested (Table 3). The chloroform extract of seed has shown a better cytotoxicity against PC3 when compared to the whole fruit; however, distilled water and methanol extracts of whole fruit exhibited better cytotoxicity compared to seed extract (Table 4). The cytotoxicity against Jurkat cell lines was found to be almost similar among all different extracts (Table 5). Both whole fruit (Figure 2) and seed extract extracts (figure not shown) exhibited good cytotoxic activity against different cancer cell lines.

The cytotoxicity was not observed in untreated cell lines; however, all the cell lines treated with curcumin (15 *μ*M) showed the cytotoxicity. The statistical analysis by one-way ANOVA followed by Dunnett's test for multiple comparisons revealed high significance at *p* < 0.0001 in comparison with control/untreated cells against all the different tested cell lines. The seed extracts from all the three solvent systems exhibited anticancer activity against HeLa cell lines; the IC_50_ values were found to be 347.55, 349.43, and 331.33 *μ*g/mL, respectively, for methanol, water, and chloroform extracts. Further, the cytotoxic activity of whole fruit extracts against HeLa cell lines revealed IC_50_ values of 264.27, 332.89, and 257.7 *μ*g/mL, respectively, for methanol, water, and chloroform extracts. The IC_50_ values of all different extracts against different cell lines are shown in Table 6. Further, the cytotoxicity effects between whole fruit and seed extracts were assessed using *t-*test (*p* < 0.05). The results showed that there was no statistically significant difference between the extracts. Overall, the seed extract was more cytotoxic to HCT116 cell line, and whole fruit extract was more cytotoxic to HeLa, PC3, and Jurkat cell lines.

## 4. Discussion

Naturally derived products are the vital source for developing novel drugs. Studies have shown that phytochemicals possess incredible health benefits and play a significant role in human disease prevention. Phytochemicals like plant secondary metabolites and antioxidants exhibit important therapeutic properties [12–14]. In general, phytocompounds are extracted from different plant sources using many ways including decoction, maceration, Soxhlet extraction, supercritical fluid extraction, microwave-aided extraction, and ultrasound-aided extraction approaches [15]. The maceration technique is widely used at the preliminary investigation level due to its simplicity and easy to use [11, 15]. Hence, maceration was used in the present study to preliminarily examine the bioactive principles and bioactivities.

The organic solvents will have a substantial impact on the total dry weight and extract yield. Many studies have evidently suggested that methanol as the best solvent to be used for recovering higher extractable phytocompounds from honeydew melon, irrespective of the plant parts [11, 16, 17]. Likewise, in the present study also, methanol solvent recovered maximum extractable elements from the muskmelon whole fruit and seed extracts. Thus, the present study results are in agreement with earlier recorded observations. The presence of substantial variations in the obtained total yield of the extract in different solvents could be correlated to polar nature of the organic solvents evaluated [11, 18].

Previously, researchers have studied cytotoxicity effect of muskmelon fruits and seeds [19, 20]. However, the research efforts were limited to only a single plant parts (seeds, peel, or whole fruit) and to single cancer cells. Moreover, the use of different solvent extracts against different cancer cells is limited. This is a first comparative study to examine the cytotoxicity effect of different solvent extracts of whole fruit and seeds against several cancer cells, which was carried out for the first time. The highest cytotoxicity observed in the extract of whole fruit could be because of higher levels of bioactive compounds present in it. Similarly, it has been documented that biological activities are directly correlated to the amount of active metabolites that occur in plant extracts [5, 8, 11].

Polyphenols are a group of valuable plant secondary metabolites known for their free radical scavenging activity in cells and protects body from many diseases, such as neuronal disease, cardiovascular diseases, cataract, and cancers [5]. Mainly, secondary metabolites of plants include polyphenolics and flavonoids, and they possess superior pharmacological activities including antimicrobial, antioxidant, anti-inflammatory, and anticancer activities [21]. The quantitative analyses of honeydew melon extracts revealed that whole fruit extract possesses the highest gallic acid and rutin contents. This could be attributed to the fact that whole fruit is being exposed to external environmental stresses as compared to seeds; it contains higher levels of polyphenols. Similarly, a previous study by Rolim and coworkers have shown that peel extract having higher levels of polyphenols compared to seeds [20].

The biological properties of honeydew melon seed and whole fruit extracts could be due to the occurrence of different phytoconstituents. The literature survey discloses the presence of several nonvolatile compounds from aqueous, methanol, and ethanol extracts of muskmelon seeds and fruit peel around the globe. To the best of our awareness, there is no single report of GC-MS analysis to characterize honeydew melon metabolites. Therefore, this study also included GC-MS analysis to identify the possible bioactive metabolites of honeydew melon extracts. The whole fruit extract was identified with numerous metabolites, including previously known compounds possessing numerous pharmacological properties. In this investigation, the superior anticancer activities exhibited by chloroform extract of whole fruit could be related to the existence of more number of biologically active compounds, such as hexadecanoic acid, undecane, and 2-dodecanol [2, 18, 22]. Further, the presence of other newly determined compounds in the extract clearly opens a new avenue for the exploration of muskmelon plant in drug discovery and other medicinal applications. Yet, more examinations are needed to identify and characterize other metabolites from muskmelon and evaluate their pharmacological importance.

## 5. Conclusion

The present study revealed the occurrence of various soluble biologically active phytocompounds in honeydew melon whole fruit and seed extracts using different solvent systems. Overall, the study evidenced the role of phytocompounds occurring in honeydew melon extracts in inhibiting the growth of several types of cancer cells. Also, polyphenol content and yield of the extract varied depending on the solvent types used for the extraction. In the chloroform extract, more soluble metabolites were evidenced, and hence, it exhibited higher anticancer activity as compared to other solvent extracts. These observations clearly support the use of whole fruit for further investigations for treating various diseases, including cancers.

## Figures and Tables

**Table 1:** The GC-MS analysis report showing the presence of different metabolites.

Sl. No.	Apex RT	Area	% area	Height	% height	Identification
1	7.83	813202.579	0.31	330482.409	0.34	Ribitol, 1,3:2,4-di-O-benzylidene
2	13.63	415974.127	0.16	164857.783	0.17	Beta-estradiol 3,17-disulfate
3	13.9	898950.604	0.35	262301.786	0.27	Malic acid
4	14.95	356696.389	0.14	121270.499	0.12	Undecane
5	15.1	1003747.7	0.39	324390.461	0.33	2,3-Dihydroxy-2-methylpentanoic acid
6	17.4	810167.007	0.31	280707.991	0.29	Mephaneine
7	18.93	421209.113	0.16	164030.915	0.17	Oxazolam
8	20.81	138570.826	0.05	58502.816	0.06	5-(Hydroxymethyl)-2-(dimethoxymethyl)furan
9	23.23	132501.875	0.05	61695.305	0.06	9-Nonadecene
10	23.43	326043.858	0.13	125731.585	0.13	Tetradecane
11	23.61	740844.025	0.28	291654.173	0.3	Glafenin
12	26.35	4846262.298	1.86	1783633.242	1.81	2,4-Di-*tert*-butylphenol
13	26.6	326311.396	0.13	122525.028	0.12	Lauric acid, methyl ester
14	28.18	845949.533	0.33	331696.785	0.34	2-Dodecanol
15	28.35	1219645.767	0.47	409647.806	0.42	Hexadecane
16	28.56	1134791.726	0.44	411097.895	0.42	Methyl 3,5-dimethoxybenzoate
17	31.22	895920.761	0.34	326795.903	0.33	Myristic acid, methyl ester
18	32.58	356138.721	0.14	154978.494	0.16	Pentadecanoic acid, methyl ester
19	32.64	754986.253	0.29	332653.706	0.34	1-Docosene
20	32.78	2052151.175	0.79	575060.411	0.58	Nonadecane
21	32.96	1498170.836	0.58	564804.031	0.57	N^1^-(*tert*-Butyldimethylsilyl)-6-nitrobenzene-1,3-diamine
22	33.36	867260.244	0.33	307142.157	0.31	Methyl 13-methyltetradecanoate
23	34.32	764655.12	0.29	253979.509	0.26	Phthalic acid, butyl isobutyl ester
24	34.66	976392.889	0.38	359128.863	0.36	Palmitic acid, methyl ester
25	34.99	4397282.814	1.69	1756158.358	1.78	(*Z*)-Methyl hexadec-11-enoate
26	35.41	77711514.61	29.89	30214884.99	30.7	Palmitic acid, methyl ester
27	36.09	466517.711	0.18	183204.419	0.19	Hexadecanoic acid
28	36.21	5520414.14	2.12	2061336.773	2.09	Dibutyl phthalate
29	36.74	997875.035	0.38	447963.602	0.46	Palmitic acid, ethyl ester
30	36.81	669635.521	0.26	300464.6	0.31	2-Octadecoxyethanol
31	38.66	71694171.07	27.58	28428235.56	28.88	Linoleic acid, methyl ester
32	38.78	41915737.09	16.12	13833942.29	14.05	Linolenic acid, methyl ester
33	38.87	3935994.727	1.51	1775814.73	1.8	13-Octadecenoic acid, methyl ester
34	39.23	13455425.91	5.18	5116611.537	5.2	Stearic acid, methyl ester
35	39.88	649279.136	0.25	224145.171	0.23	Linoleic acid ethyl ester
36	42.65	1173609.722	0.45	447888.354	0.46	Eicosanoic acid, methyl ester
37	44.62	1960767.037	0.75	1100376.213	1.12	Docosanoic acid, methyl ester

**Table 2:** Effect of phytoextracts on cancer cell line, HeLa.

Drug/phytoextracts	Concentration	Percentage cell viability		
Control/untreated cell lines	—	100 ± 0.005		
Curcumin	15 *μ*M	49.85±0.05^∗∗∗∗^		

		Methanol extract	Distilled water extract	Chloroform extract
Whole fruit	50 *μ*g/mL	89.80±0.007^∗∗∗∗^	91.40±0.006^∗∗∗∗^	86.12±0.004^∗∗∗∗^
Seeds		86.02±0.004^∗∗∗∗^	84.36±0.004^∗∗∗∗^	93.68±0.006^∗∗∗∗^
Whole fruit	150 *μ*g/mL	75.25±0.005^∗∗∗∗^	76.19±0.001^∗∗∗∗^	72.46±0.005^∗∗∗∗^
Seeds		77.95±0.002^∗∗∗∗^	77.95±0.002^∗∗∗∗^	76.08±0.004^∗∗∗∗^
Whole fruit	250 *μ*g/mL	46.27±0.003^∗∗∗∗^	64.18±0.001^∗∗∗∗^	51.35±0.005^∗∗∗∗^
Seeds		63.04±0.003^∗∗∗∗^	61.07±0.005^∗∗∗∗^	56.02±0.02^∗∗∗∗^
Whole fruit	350 *μ*g/mL	32.60±0.007^∗∗∗∗^	54.86±0.004^∗∗∗∗^	32.40±0.005^∗∗∗∗^
Seeds		54.76±0.002^∗∗∗∗^	50.51±0.005^∗∗∗∗^	43.78±0.003^∗∗∗∗^
Whole fruit	450 *μ*g/mL	18.32±0.004^∗∗∗∗^	25.87±0.004^∗∗∗∗^	14.59±0.005^∗∗∗∗^
Seeds		32.09±0.003^∗∗∗∗^	36.12±0.002^∗∗∗∗^	38.01±0.004^∗∗∗∗^

Comparative cytotoxic effect of different solvent extracts of honeydew melon on HeLa cell lines after 24 h of incubation. Data is expressed as the mean ± SD (each treatment, *n* = 3). ∗∗∗∗ indicates *p* < 0.0001 in comparison with control/untreated cells by one-way ANOVA followed by Dunnett's test for multiple comparisons.

**Table 3:** Effect of phytoextracts on cancer cell line, HCT116.

Drug/phytoextracts	Concentration	Percentage cell viability		
Control/untreated cell lines	—	100 ± 0.006		
Curcumin	15 *μ*M	50.63±0.003^∗∗∗∗^		

		Methanol extract	Distilled water extract	Chloroform extract
Whole fruit	50 *μ*g/mL	80.15±0.007^∗∗∗∗^	84.27±0.001^∗∗∗∗^	89.25±0.01^∗∗∗∗^
Seeds		80.35±0.004^∗∗∗∗^	83.94±0.006^∗∗∗∗^	80.40±0.007^∗∗∗∗^
Whole fruit	150 *μ*g/mL	70.53±0.003^∗∗∗∗^	66.75±0.004^∗∗∗∗^	73.98±0.003^∗∗∗∗^
Seeds		68.81±0.007^∗∗∗∗^	69.87±0.006^∗∗∗∗^	70.40±0.01^∗∗∗∗^
Whole fruit	250 *μ*g/mL	49.30±0.007^∗∗∗∗^	56.93±0.004^∗∗∗∗^	60.45±0.007^∗∗∗∗^
Seeds		48.50±0.007^∗∗∗∗^	58.46±0.002^∗∗∗∗^	47.37±0.002^∗∗∗∗^
Whole fruit	350 *μ*g/mL	31.25±0.007^∗∗∗∗^	46.25±0.01^∗∗∗∗^	42.01±0.007^∗∗∗∗^
Seeds		37.35±0.003^∗∗∗∗^	47.40±0.01^∗∗∗∗^	30.12±0.01^∗∗∗∗^
Whole fruit	450 *μ*g/mL	28.46±0.003^∗∗∗∗^	16.72±0.002^∗∗∗∗^	27.07±0.004^∗∗∗∗^
Seeds		22.49±0.01^∗∗∗∗^	27.14±0.007^∗∗∗∗^	18.97±0.004^∗∗∗∗^

Comparative cytotoxic effect of different solvent extracts of honeydew melon on HCT116 cell lines after 24 h of incubation. Data is expressed as the mean ± SD (each treatment, *n* = 3). ∗∗∗∗ indicates *p* < 0.0001 in comparison with control/untreated cells by one-way ANOVA followed by Dunnett's test for multiple comparisons.

**Table 4:** Effect of honeydew melon phytoextracts on cancer cell line, PC3.

Drug/phytoextracts	Concentration	Percentage cell viability		
Control/untreated cell lines	—	100 ± 0.008		
Curcumin	15 *μ*M	49.08±0.002^∗∗∗∗^		

		Methanol extract	Distilled water extract	Chloroform extract
Whole fruit	50 *μ*g/mL	96.77±0.01^∗∗∗∗^	86.33±0.004^∗∗∗∗^	89.00±0.007^∗∗∗∗^
Seeds		96.89±0.005^∗∗∗∗^	98.32±0.002^∗∗∗∗^	89.56±0.002^∗∗∗∗^
Whole fruit	150 *μ*g/mL	80.47±0.002^∗∗∗∗^	75.77±0.002^∗∗∗∗^	77.45±0.01^∗∗∗∗^
Seeds		91.63±0.005^∗∗∗∗^	90.43±0.009^∗∗∗∗^	75.85±0.01^∗∗∗∗^
Whole fruit	250 *μ*g/mL	70.35±0.004^∗∗∗∗^	62.70±0.007^∗∗∗∗^	65.49±0.001^∗∗∗∗^
Seeds		75.53±0.01^∗∗∗∗^	69.08±0.002^∗∗∗∗^	56.77±0.01^∗∗∗∗^
Whole fruit	350 *μ*g/mL	58.80±0.002^∗∗∗∗^	50.91±0.003^∗∗∗∗^	52.66±0.004^∗∗∗∗^
Seeds		64.38±0.004^∗∗∗∗^	56.65±0.003^∗∗∗∗^	41.75±0.001^∗∗∗∗^
Whole fruit	450 *μ*g/mL	34.66±0.003^∗∗∗∗^	46.62±0.009^∗∗∗∗^	36.81±0.005^∗∗∗∗^
Seeds		44.22±0.006^∗∗∗∗^	39.92±0.01^∗∗∗∗^	31.39±0.007^∗∗∗∗^

Comparative cytotoxic effect of different solvent extracts of honeydew melon on PC3 cell lines after 24 h of incubation. Data is expressed as the mean ± SD (each treatment, *n* = 3). ∗∗∗∗ indicates *p* < 0.0001 in comparison with control/untreated cells by one-way ANOVA followed by Dunnett's test for multiple comparisons.

**Table 5:** Effect of honeydew melon phytoextracts on cancer cell line, Jurkat.

Drug/phytoextracts	Concentration	Percentage cell viability		
Control/untreated cell lines	—	100 ± 0.009		
Curcumin	15 *μ*M	43.10±0.007^∗∗∗∗^		

		Methanol extract	Distilled water extract	Chloroform extract
Whole fruit	50 *μ*g/mL	79.48±0.006^∗∗∗∗^	90.77±0.007^∗∗∗∗^	87.81±0.001^∗∗∗∗^
Seeds		87.45±0.009^∗∗∗∗^	73.63±0.002^∗∗∗∗^	88.62±0.004^∗∗∗∗^
Whole fruit	150 *μ*g/mL	68.27±0.002^∗∗∗∗^	85.03±0.004^∗∗∗∗^	67.74±0.004^∗∗∗∗^
Seeds		71.59±0.002^∗∗∗∗^	66.21±0.002^∗∗∗∗^	74.55±0.001^∗∗∗∗^
Whole fruit	250 *μ*g/mL	46.32±0.004^∗∗∗∗^	69.62±0.006^∗∗∗∗^	52.68±0.004^∗∗∗∗^
Seeds		58.06±0.004^∗∗∗∗^	56.36±0.003^∗∗∗∗^	47.31±0.004^∗∗∗∗^
Whole fruit	350 *μ*g/mL	34.1±0.003^∗∗∗∗^	52.86±0.005^∗∗∗∗^	29.65±0.003^∗∗∗∗^
Seeds		43.99±0.003^∗∗∗∗^	47.04±0.002^∗∗∗∗^	32.61±0.009^∗∗∗∗^
Whole fruit	450 *μ*g/mL	16.68±0.002^∗∗∗∗^	38.08±0.006^∗∗∗∗^	11.55±0.003^∗∗∗∗^
Seeds		27.41±0.008^∗∗∗∗^	28.13±0.005^∗∗∗∗^	16.12±0.008^∗∗∗∗^

Comparative cytotoxic effect of different solvent extracts of honeydew melon on Jurkat cell lines after 24 h of incubation. Data is expressed as the mean ± SD (each treatment, *n* = 3). ∗∗∗∗ indicates *p* < 0.0001 in comparison with control/untreated cells by one-way ANOVA followed by Dunnett's test for multiple comparisons.

**Table 6:** Cytotoxic activity of different phytoextracts against different cancer cell lines and IC_50_ values.

Phytoextracts		IC_50_ values			
Fruit part	Solvent	HeLa	HCT116	PC3	Jurkat
Seed	Methanol	347.55 *μ*g/mL	259.25 *μ*g/mL	436.36 *μ*g/mL	303.54 *μ*g/mL
	Water	349.43 *μ*g/mL	304.42 *μ*g/mL	390.0 *μ*g/mL	293.13 *μ*g/mL
	Chloroform	331.33 *μ*g/mL	246.93 *μ*g/mL	319.8 *μ*g/mL	261.13 *μ*g/mL

Whole fruit	Methanol	264.27 *μ*g/mL	264.86 *μ*g/mL	376.92 *μ*g/mL	243.47 *μ*g/mL
	Water	332.89 *μ*g/mL	277.93 *μ*g/mL	373.09 *μ*g/mL	376.64 *μ*g/mL
	Chloroform	257.70 *μ*g/mL	290.32 *μ*g/mL	341.01 *μ*g/mL	250.21 *μ*g/mL

## Data Availability

Not applicable.
